# Complete Genome Sequence of WM99c, an Antibiotic-Resistant Acinetobacter baumannii Global Clone 2 (GC2) Strain Representing an Australian GC2 Lineage

**DOI:** 10.1128/MRA.01199-18

**Published:** 2018-12-06

**Authors:** Steven J. Nigro, Ryan Wick, Kathryn E. Holt, Ruth M. Hall

**Affiliations:** a School of Life and Environmental Sciences, University of Sydney, Sydney, Australia; b Centre for Systems Genomics, University of Melbourne, Melbourne, Australia; c Department of Biochemistry and Molecular Biology, Bio21 Molecular Science and Biotechnology Institute, University of Melbourne, Melbourne, Australia; Indiana University, Bloomington

## Abstract

The extensively antibiotic-resistant Acinetobacter baumannii isolate WM99c recovered in Sydney, Australia, in 1999 is an early representative of a distinct lineage of global clone 2 (GC2) seen on the east coast of Australia. We present the complete 4.121-Mbp genome sequence (chromosome plus 2 plasmids), generated via long-read sequencing (PacBio).

## ANNOUNCEMENT

The Acinetobacter baumannii isolate WM99c was collected in 1999 at Westmead Hospital in Sydney, Australia (1), and is one of the earliest global clone 2 (GC2) isolates recorded in Australia (2, 3). It is resistant to several current antibiotics, including imipenem, meropenem, ceftazidime, cefotaxime, fluoroquinolones, ciprofloxacin, and levofloxacin, as well as older antibiotics, including sulfonamides, tetracycline, minocycline, and the aminoglycosides gentamicin, kanamycin, and neomycin (4). It is also resistant to tigecycline (5). It was previously shown to be representative of a distinct GC2 lineage that has the *oxa23* carbapenem resistance gene in transposon Tn*2006* and an ISAba17 in the AbGRI1-2 variant (formerly Tn*6167* [6]) of the AbGRI1 resistance island, which is located in the *comM* gene (4). This variant has been seen only in isolates from hospitals on the east coast of Australia (3). A characteristic variant of the AbGRI2 resistance island, AbGRI2-1, is also present in the chromosome (4). Third-generation cephalosporin resistance is due to increased transcription of the *ampC* gene from an upstream ISAba1, and fluoroquinolone resistance arose via mutations in *gyrA* and *parC*.

Whole-cell DNA was prepared (7) from cells grown in LB, quality controlled for length using agarose gel electrophoresis, and quantified with the Qubit (Invitrogen) system. The DNA was subjected to library preparation and sequencing on 2 PacBio single-molecule real-time (SMRT) cells (chemistry version C2-P4) at DNA Link (South Korea). A total of 164,293 reads were obtained with an average length of 12,816 bp and average quality of 0.823. The PacBio reads (2.1 Gbp; SRA accession number SRR8162697) were retrieved from the raw data using PacBio’s SMRT Analysis software and quality control (QC) filters of a PacBio quality score of 85 or greater, and a length of 13 kbp or greater was used to take the read set to a manageable size of 300 Mbp. These reads were combined with available Illumina HiSeq data (SRA accession number ERR110084) with Unicycler version 0.4.4 (8) with default parameters, and the resulting assembly was polished with both read sets with unicycler-polish.

The assembled contiguous circular sequence of the chromosome was 4,016,817 bp and had a 39.12% GC content. Two plasmid contigs represented pWM99c-1 (11 kb), which is identical to pD72-1 (GenBank accession number KM051986), and pWM99c-2 (110 kb), which is identical to pC20-3 (9) and >99.9% identical (6 single-base-pair differences) to pABTJ2 (GenBank accession number CP004359). A 13.3-kb integrative element encoding a site-specific tyrosine recombinase was detected in the excised free circular form as well as in the chromosome (bp 992828 to 1006173). Protein coding, rRNA, and tRNA genes were annotated with Prokka version 1.12 (10), and the antibiotic resistance regions, transposons, insertion sequences, and plasmids were annotated manually. Polysaccharide biosynthesis loci for capsule (KL) and outer core of lipooligosaccharide (OCL) were annotated according to published nomenclature (11). Four potential integrated phage genomes of 52.6 kb, 49.5 kb, 37 bp, and 36.1 kb were identified with PHASTER (12).

WM99c belongs to sequence type 208 (ST208) (Oxford scheme; https://pubmlst.org/abaumannii/) and carries the KL2 capsule cluster and the OCL1 lipooligosaccharide outer core cluster (11). In addition to the IS*26* copies in AbGRI2-1, the chromosome includes 20 copies of the insertion sequence ISAba1, including 4 associated with expression of antibiotic resistance genes, namely, the 2 that are part of Tn*2006* and the single copies upstream of the *ampC* and *sul2* resistance genes. Two copies each of ISAba17 and ISAba22 and 1 of ISAba26 were also detected using ISfinder (https://isfinder.biotoul.fr/).

The genome sequence of WM99c will underpin studies of the origin and evolution of the unique GC2 lineage found in hospitals on the east coast of Australia.

### Data availability.

The complete genome sequence of the Acinetobacter baumannii isolate WM99c has been deposited in DDBJ/ENA/GenBank under the accession numbers CP031743 (chromosome), CP031745 (pWM99c-1), and CP031744 (pWM99c-2). The versions described in this paper are the first versions, CP031743.1 to CP031745.1. The PacBio reads have been deposited in the SRA under accession number SRR8162697. Illumina HiSeq data are available under SRA accession number ERR110084.
